# Knowledge, attitude and practice regarding eye complications and care among Omani persons with diabetes - A cross sectional study

**DOI:** 10.4103/0974-620X.64228

**Published:** 2010

**Authors:** Rajiv Khandekar, Saleh Al Harby, Harith Al Harthy, Jawad Al Lawatti

**Affiliations:** Eye and Ear Health Care, Department of Non-communicable Disease Control, Oman; 1 Director General of Health Affairs, Ministry of Health, Oman

**Keywords:** Diabetes, diabetic retinopathy, KAP, Oman

## Abstract

**Purpose:**

We present the level of Knowledge, Attitude and Practice (KAP) among diabetic patients regarding eye complications and their care.

**Materials and Methods:**

A cross sectional study was conducted in 2008 at seven regions of Oman. Arabic speaking nurses interviewed diabetics at clinics. They used a closed ended questionnaire with 15 questions. The responses were analyzed and the KAP were grouped into excellent (>80%), good (60 to 79%), average (40 to 59%), poor (20 to 39%) and very poor (<20%). They were also compared among epidemiologic variants.

**Result:**

Of the 750 participants, ‘Excellent’, grade of knowledge about diagnosis and eye care was present in 547 (72.9%) and 135 (18%) persons respectively. The ‘excellent’ grade of attitude about eye involvement and eye care was found in 135 (18%) and 224 (29.9%) participants. The practice for undergoing eye check up and accepting treatment was of ’excellent’ grade in 390 (52%) and 594 (79.2%) respectively. Age (OR = 0.98), Sharqiya region (OR = 25) and ‘5 to 9’ duration of DM (OR = 2.1) were associated with the knowledge. ‘<1 year’ duration (OR = 0.3) and Dhakhiliya region (OR = 39) were associated with the attitude while ‘5 to 9 year’ duration (OR = 3.4) was associated with better practices.

**Conclusions:**

Knowledge about eye complications and care is satisfactory among persons with diabetes. However, levels of attitude and practice were less than desired and should be improved. This could strengthen program approach for early detection and care of eye complications of diabetes in Oman.

## Introduction

Diabetic Retinopathy (DR) is a priority blinding disease and is now included in the disease control strategy of ‘VISION 2020’ initiative.[1,2] The WHO has published guidelines for program approach to address DR.[3] I spite of rapid technological advances in screening and management of DR, primary prevention still remains to be the only feasible approach in many developing countries with competing demands. To delay the progress of eye complications, in addition to the periodic eye examination and timely interventions, it is crucial that the patients with diabetes judicially control their hypertension, blood sugar and lipid levels.[4] To prepare effective method of health promotion about diabetes, baseline information about knowledge, attitude and practice regarding eye complication and eye care of diabetics is crucial. Such studies were conducted in India,[5] Australia[6] and Italy,[7] however to the best of our knowledge; no such study has been undertaken in the Middle East.

In Oman, diabetes is of epidemic proportion.[8] Due to improved health services and accessible as well as affordable renal dialysis facilities, diabetic patients are surviving longer. Eye complications that are known to be associated with the longer duration of diabetes cause visual impairment. With nearly 70,000 registered diabetics in Oman, counseling them for primary prevention is a daunting task.[9] Promotion of healthy life style to the community and counseling of patients with diabetes have many components. We conducted this study to determine present level of knowledge, attitude and practice about eye complications and care for diabetes and propose health promotion polices accordingly.

## Materials and Methods

The ethical and research committee of the Ministry of Health, Oman reviewed and approved the study proposal. This study was conducted in 2008.

This was a health institution based cross sectional study. Citizen with diabetes that were registered in seven northern and central regions of Oman were our study population. We calculated the sample size with precision at regional level. The registered diabetics ranged from 329 to 11,200 in different regions. We assumed that excellent grade of knowledge, attitude and practice will be in 40% of the diabetes patients. To conduct study with 95% confidence interval and acceptable error margin of 15%, we needed 41 patients in each region. To compensate the loss due to non participation, we increased the sample by 20%. Thus minimum sample in each region required was 50 persons. A list of health institutions with number of registered diabetics was prepared for each region. Based on unit of 100 diabetics, a health institution was given option of being selected in the study. By using random table in Microsoft XL software, we selected 10 units in each region. On a randomly selected day of a week, field investigators visited this institution. The first five patients in sequence were enrolled for the study.

One health educator and a regional eye health care supervisor were teamed and seven such teams were made. They informed health administrators of the region about time and purpose of the study. Written consents of health administrators were obtained to conduct study in their respective regions. Verbal informed consent of the enrolled participants was obtained.

A close ended questionnaire was used to collect the responses [Table 1]. They comprised of five questions each on the knowledge about eye complications of diabetes and eye care. Three attitude related questions on primary prevention and eye care of diabetic retinopathy were included. Finally three questions regarding practice being followed by participant to take care of his/her eyes were asked. Without prompting of investigator or relative accompanying the participant, responses were collected. Personal information like age, sex, wilayat/region of residence, duration of diabetes and diabetes registration number were recorded. Five graded responses were used for each question. The correct responses of each question were determined by the study investigators prior to the study. If the response of participants to these questions matched with gold standard, it was considered as correct and 10 points for that response was awarded. For totally wrong answer, minus 10 points were given. For equivocal response ’0’ point was designated. The total points of knowledge, attitude and practice related questions were regrouped in four categories. Person with 75% to 100% score was considered to have ’excellent’ grade of response. If the score was 50% to 74%, it was considered as satisfactory. Persons scoring 25% to 49% and 0% to 24% were grouped into poor and very poor grades respectively.

**Table 1:** Responses of different questions related to knowledge, attitude and practice among patients with diabetes in Oman (KAP Diabetes 2008)

*Questions*		*Responses (n = 750)*				
		*Fully agree*	*Agree*	*Can’t decide*	*Disagree*	*Fully disagree*
1.1	Diabetes can damage eyesight	456	244	41	7	2
1.2	Retina is the main part of eyes that gets damaged in diabetes	336	284	111	17	2
1.3	Eye doctor will examine eyes using special equipment to find effects of diabetes	380	325	30	13	2
1.4	Timely treatment can prevent/ delay damage due to diabetes in eyes	443	276	25	3	3
1.5	Control Blood sugar and lipids makes eye treatment effective	256	264	68	118	44
1.6.	In diabetes, one eye may be affected first followed by the other eye	274	321	127	24	4
1.7	Children with diabetes also have risk of eye complications	205	229	256	44	16
1.8	An eye with diabetic retinopathy if successfully treated with the laser, it does not need laser treatment again	66	114	334	202	34
1.9	Laser treatment of diabetes is painful	40	104	470	106	30
1.10	If vision is damaged due to diabetes, use of ‘low vision’ aids helps in daily work	258	291	87	106	8
2.1	If my vision is good, my eyes are not affected. So I do not need annual eye testing.	87	135	19	293	216
2.2	The information on eye problems due to diabetes should be given by eye doctor only	185	356	35	151	23
2.3	If I am taking eye treatment, I need not worry about controlling my sugar and lipid	55	131	43	296	225
2.4	If my eye is treated with laser once, I don’t need laser treatment again in that eye.	43	72	305	241	89
2.5	Patients with diabetes often waste their time and money in eye check ups as most of the time eyes of diabetics are normal	117	110	91	295	137
2.6	One should not be treated with laser as it is very painful	36	57	419	182	56
3.1	I go to eye doctor regularly as advised by my family doctor	390	312	18	22	8
3.2	I control my blood sugar and lipid even if eye complication is being treated.	377	334	16	20	3
3.3	Staff in eye unit counseled me about prevention and treatment for eye complications	290	389	20	34	17
3.4	My vision due to complications of diabetes is less. Hence I am using low vision devices	272	334	45	83	16

We used pretested data collection form. The data from these forms was transformed on spreadsheet using EPI Data software.[10] We used Statistical Package for Social Studies (SPSS 11) for the analysis. Univariate analysis was conducted by parametric method. We calculated frequencies and percentage proportions. To determine the predictors of ‘satisfactory’ (excellent + good) grade of knowledge, attitude and practice for eye care among persons with diabetes, we conducted multi-nominal logistic regression analysis. Age, gender, region of residence and duration of diabetes were the independent variables in the step in method of model.

Those with poor level of belief and practice were counseled about the benefits of primary prevention and laser treatment of DR. The identity of participants was de-linked from other information to maintain confidentiality.

## Results

We interviewed 750 persons with diabetes in seven regions. More than half of the participants were of 40 to 59 years of age and had diabetes since last five years. Female participants were more compared to male participants.

The level of knowledge, attitude and practice regarding eye complications and eye care is given in Table 2. Knowledge of eye complications of diabetes was of excellent grade in 72.9% (95% CI 69.2 – 76.6). However the knowledge of eye treatment of diabetic retinopathy was of excellent grade in 18% (95% CI 11.5 – 24.5) only. The attitude for eye screening was very positive in 18% (95% CI 11.5 – 24.5) only. The attitude was very positive for laser treatment of DR in 29.9% (95% CI 23.9 – 35.9). The responses regarding practice followed for eye screening was of excellent grade in 52% (95% CI 47 – 57). But 79.2% (95% CI 75.9 – 82.5) patients practiced for laser treatment.

**Table 2:** Knowledge, attitude and practice of diabetes patients (KAP Diabetes 2008)

	*Excellent*		*Good*		*Poor*		*Very poor*	
	#	%	#	%	#	%	#	%
Knowledge of eye complications of diabetes	547	72.9	191	25.5	12	1.6	0	0.0
Knowledge of eye treatment in diabetes	135	18.0	237	31.6	325	43.3	53	7.1
Attitude for eye check up	135	18.0	237	31.6	325	43.3	53	7.1
Attitude for management of diabetic retinopathy (DR)	224	29.9	288	38.4	212	28.3	26	3.5
Practice for periodic eye checks	390	52.0	312	41.6	40	5.3	8	1.1
Practice for laser treatment of DR	594	79.2	123	16.4	32	4.3	1	0.1

Excellent = cumulative point >75; Good = cumulative points 50 to 74; Poor = cumulative points 25 to 49; Very poor = cumulative points less than 25

The predictors of satisfactory level of Knowledge, attitude and Practice were evaluated by binominal regression analysis [Table 3]. The analysis suggests that the level of knowledge could be predicted by knowing the age, duration of DM and the area of residence (Region) of the person with diabetes. The level of positive attitude for eye care among persons with diabetes could be predicted if duration of diabetes, age and region of residence are known. The satisfactory practice of eye care was positively associated with ‘5 to 9 years’ duration of diabetes.

**Table 3:** Influence of variant on ‘Satisfactory’* grade of KAP among diabetes patients (KAP Diabetes 2008)

		*Number of participants*	*Knowledge*			*Attitude*			*Practice*		
			*Adjusted odd’s ratio*	*95% confidence interval*	*P value*	*Adjusted odd’s ratio*	*95% confidence interval*	*P value*	*Adjusted odd’s ratio*	*95% confidence interval*	*P value*
Gender	Male	313	1.27	0.92 – 1.74	0.14	1.15	0.81 – 1.6	0.41	1.00	0.98 – 1.11	0.89
	Female	437	1								
Age			0.98	0.96 – 0.99	0.001	0.99	0.96 – 1.01	0.34	1.17	0.79 – 1.74	0.42
Duration of diabetes	<1 years	104	2.1	0.94 – 4.8	0.07	0.3	0.11 – 0.83	0.02	1.19	0.5 – 2.8	0.69
	1 to 4.9	274	1.6	0.78 – 3.5	0.19	0.39	0.15 – 1.02	0.05	2.5	1.14 – 5.6	0.02
	5 to 9.9	236	2.01	1.03 – 4.5	0.04	0.32	0.12 – 0.83	0.02	3.4	1.5 – 7.6	0.003
	10 to 14.9	90	1.5	0.64 – 2.04	0.36	0.28	0.11 – 0.8	0.02	2.2	0.93 – 5.3	0.07
	15 +	40	1			1			1		
	missing	6									
Type of diabetes	Type I	135	0.94	0. 60– 1.4	0.77	1.09	0.68 – 1.70	0.72	0.99	0.58 – 1.7	0.98
	Type II	615	1			1			1		
Region	Muscat	101	9.2	3.8 – 21.9	<0.01	52	14 – 183	<0.01	0.87	0.38 – 2.01	0.76
	Dhakhiliya	100	2.6	1.1 – 6.3	0.03	39.1	11.1 – 137	<0.01	1.2	0.5 – 2.8	0.72
	N Sharqiya	49	25.2	8.7 –72.7	<0.01	58	15 – 228	<0.01	6.6	1.3 – 31.7	0.02
	S Sharqiya	100	5.2	2.2 – 12.3	0.0002	63	7.7 – 228	<0.01	1.5	0.62 – 3.6	0.36
	N Batinah	200	3.6	1.6 – 8.1	0.002	26.5	7.9 – 88.6	<0.01	1.1	0.5 – 2.3	0.85
	S Batinah	150	6.25	2.7 – 14.4	<0.01	10.1	3 – 31	0.002	2.1	0.92 – 4.9	0.07
	Dhahira	50	1			1			1		
Constant			−1.2			−1.4			0.26		

* Satisfactory = excellent + good

The responses to each questions related to Knowledge, Attitude and Practice by participants is given in Table 1.

Our study results were compared to the similar studies conducted and published in literature [Table 4].[11–22]

**Table 4:** Level of KAP in persons with diabetes in Oman and other studies (KAP Diabetes 2008)

*Topic*	*Year*	*Country*	*Participants*	*Level (%)*	*Reference*
Knowledge of eye diseases in persons with diabetes of >1 year	2008	USA	204	52	11
Practice of eye examination					
Knowledge of blindness due to diabetes	2008	Myanmar	480	86	12
Visited ophthalmologist				57	
Practice of undergoing eye examination	2007	Tanzania	316	59.1	13
Awareness of eye and diabetes Attitude towards eye complications	2004	UK	500	Racial variation	14
Aware about eye complications	2003	Japan	1333	98	15
Underwent eye examination				69.5	
Followed vision care guidelines	2001	USA	2308	65	16
Knowledge in suburban women with diabetes	1997	USA	150	83	17
Faulty belief of symptoms of retinopathy	1997	USA	104	87	18
Risk of diabetic retinopathy	2004	India	204	50	19
Laser is treatment for Diabetic Retinopathy				10	
Visited ophthalmologists				43.5	
Ocular effect of diabetes	2003	Australia	500	96	20
Consulted ophthalmologist				63.6	
Attitude for eye check up in patients on insulin	1997	Australia	229	75	21
Patient had test for glycosylated hemoglobin	1997	USA	295	50	22

## Discussion

Human behavior of ignoring early signs of chronic disease and then in late stages to run from pillar to the post needs to be modified if organized approach is to be adopted for avoiding debilitating complications of diseases like diabetes. Diabetes is a lifelong disease and is in epidemic proportion in most of the countries. To prevent mortality and severe complications of diabetes, the person suffering from diabetes has to comply with the advices for prevention and timely care. But, these are often poorly understood by the patients and their relatives. In such situation, improving their KAP towards eye care is vital to achieve VISION 2020 goals for eliminating avoidable blindness.[2] Baseline information about KAP will assist us in strengthening the policies for advocacy. Therefore our study outcomes are crucial for revising the policies of eye care in the 8^th^ Five Year Plan of Oman (2010 – 2015).

Our study revealed that among persons with diabetes in Oman, the level of knowledge was low and ‘positive’ attitude towards eye care for diabetes was also lower than desired. In contrast, the satisfactory level of practice (screening and treatment) was noted in very high proportions of participants. High level of practice and low level of knowledge is a matter of concern. It needs to be confirmed by a longitudinal study. By knowing ‘5 to 10 years’ duration of diabetes, the area of residence (region) of the participant one could determine the level of KAP. The information of gender and type of diabetes of the participants were not useful in predicting level of KAP as element of chance observation could not be ruled out.

Large and representative sample and quality assurance procedures enabled our study to generate information that could be extrapolated to the Omani population with diabetes as registered with health institutions in each region of Oman.

The excellent grade of knowledge about eye complications due to diabetes that was found (72%) in our study was much less than 98% observed in Japan[15] and 96% in Australia.[20] High literacy rates in these countries compared to the elderly and illiterate patients with diabetes in Oman^*^ and proactive counseling in these other countries could be the reasons for this difference. However, our study results were better than 50% reported in India,[19] 52% in USA.[11] Perhaps difference in grading system to define level of knowledge could be also responsible for lower level compared to our study.

The level of knowledge in relation to treatment for eye complications of diabetes was of excellent grade in 18% of participants in our study. The study in India also reported that only 10% of the persons with diabetes knew about laser treatment of diabetes. While formulating health promotion strategy the difference in knowledge about eye complications and modes of prevention and treatment should be noted for better impact of health education material.

In spite of having high level of knowledge about eye involvement in diabetes, positive attitude of periodic eye check up in 18% and eye care in 30% suggests there is a long way to go for effectively change their health behavior in Oman. In a study in UK, the Asian population was found to have less positive attitude for eye care compared to Caucasians.[14] This observed racial difference in attitude could be the reason for the level of attitude found in Omani community. However, 75% of patients with diabetes had positive attitude for eye check up in Australia.[21] As these patients were treated with insulin, their interaction with health staff will be more frequent. In addition, they might also have faced crisis like hypoglycemia or hyperglycemia. This could explain high rate of positive attitude for health check including eye examination.

In our study, the ‘excellent’ grade of practice for eye check up was in 50% of participants and 80% for those in need of laser treatment. This is unusual as the level of knowledge regarding eye check up was high and the level of knowledge for eye treatment was low. Perhaps access to free health services at primary health care and eye care units to all Omani population might be responsible for better practice among participants even though the level of knowledge was less than desired. The issue needs further study to confirm the paradoxical levels of KAP in our study and identify the barriers of poor attitude and better practice.

While comparing our study results with those mentioned in the literature, we noted that 57% of participants had visited ophthalmologists.[12] Vision care guidelines were properly followed in 65% of participates in USA,[16] 59% in Tanzania[13] and 70% in Japan.[15]

We also attempted to determine factors associated with ‘satisfactory’ grades of knowledge, positive attitude and better practices. Age, regions and duration were associated to the knowledge. Longer duration and region were associated to the attitude while longer duration was associated to better practices. Age and gender were not associated to the KAP among persons with diabetes in our study. This was also observed in Myanmar study.[12] Thus age and gender specific intervention strategy may not be required to improve the KAP of patients with diabetes in Oman.

In our study there were few limitations. The data of Dhofar region was misplaced and lost. Hence findings of northern regions should be extrapolated to the entire population of Oman with caution. The questionnaire used in present study was different from Knowledge and Practices Diabetes Questionnaire (KPDQ) that was used in Italy.[23]

This baseline information about knowledge, attitude and practice regarding eye care among persons with diabetes should be compared with outcomes of similar study after campaign for health promotion is carried out. While planning the health promotion, weak areas of knowledge mainly for intervention should be focused intensely.
